# 100K Pathogen Genome Project: 306 *Listeria* Draft Genome Sequences for Food Safety and Public Health

**DOI:** 10.1128/genomeA.00967-16

**Published:** 2017-02-09

**Authors:** Poyin Chen, Nguyet Kong, Bihua Huang, Kao Thao, Whitney Ng, Dylan Bobby Storey, Narine Arabyan, Azarene Foutouhi, Soraya Foutouhi, Bart C. Weimer

**Affiliations:** 100K Pathogen Genome Project, School of Veterinary Medicine, UC Davis, Davis, California, USA

## Abstract

*Listeria monocytogenes* is a food-associated bacterium that is responsible for food-related illnesses worldwide. This is the initial public release of 306 *L. monocytogenes* genome sequences as part of the 100K Pathogen Genome Project. These isolates represent global genomic diversity in *L. monocytogenes*.

## GENOME ANNOUNCEMENT

*Listeria monocytogenes* is a foodborne pathogen prevalent in environmental and food matrices, including soil, fruit, and deli meats (1). This organism encompasses 13 serotypes, of which serotypes 1/2a, 1/2b, and 4b are most commonly associated with disease, including gastroenteritis and abortions (2). Listeriosis accounts for less than 1% of foodborne illnesses worldwide yet carries the highest foodborne mortality rate (16%), with immunocompromised, pregnant, young, and old individuals at risk (3). When contracted during gestation, listeriosis results in miscarriage, stillbirths, or premature labor leading to infant mortality (4). Transmission is facilitated by its capability to proliferate at low temperatures and to tolerate heat, acid stress, and salt stress, necessitating *L. monocytogenes*-specific sanitization protocols in processing plants (5–7).

Recent years have seen a spike in multistate outbreaks of listeriosis due to food contamination, including in deli meats, dairy, hummus, cantaloupe, and stone fruit, leading to recalls of contaminated products and resulting in millions of dollars in lost revenue (3). Genomic comparison of these outbreak strains with environmental isolates via whole-genome sequencing will shed light on the genetic factors that potentiate *L. monocytogenes* virulence.

The 100K Pathogen Genome Project is large-scale sequencing effort focused on producing genomes from various sources worldwide that represent isolates from the environment, plants, animals, and humans. This consortium coordinates isolate submission and DNA sequencing for pathogens of relevance to animal and human health associated with zoonotic and foodborne diseases. Sequences associated with the consortium can be found at the 100K Project website (http://100kgenomes.org). We sequenced *Listeria* genomes from around the world and from diverse isolation sources to better understand the genomic diversity of the genus. This also serves as a reference repository of genetic information for this group of organisms.

All cultures were shipped to the Weimer laboratory (UC Davis, Davis, CA), checked for purity, frozen, grown on 1.5% brain heart infusion (BHI) agar (Difco, Franklin Lakes, NJ), and lysed (8); genomic DNA was isolated (gDNA) (9), checked for quality (10), and fragmented (11). Libraries were 250 to 500 bp (12, 13), indexed (96/lane), and sequenced (Illumina HiSeq 2000 [PE100] [BGI@UCD; Sacramento, CA]). Paired-end reads were assembled using ABySS 1.5.2 using *k* = 64 (14). To date, the 100K Pathogen Genome Project has produced sequences for 313 different isolates of *Listeria*. In this release, the 100K Project produced 401 Mb of sequence, with an average calculated sequencing depth of 135× per genome.

### Accession number(s).

Sequences can be found at the 100K Project BioProject PRJNA203445 in the Sequence Read Archive (http://www.ncbi.nlm.nih.gov/sra) (Table 1).

**TABLE 1:** Summary statistics for samples identified as *Listeria monocytogenes*

GenBank accession no.	SRA accession no.	Isolate name	Avg depth of sequence (×)	No. of contigs	Genome size (bp)	Total no. of genes	Total no. of CDSs^a^
MJSI00000000	SRR1814333	BCW_2352	53	30	3,012,101	3,034	2,965
MJSJ00000000	SRR1814334	BCW_2353	52	20	2,888,472	2,869	2,810
MJSK00000000	SRR1814335	BCW_2354	51	22	2,991,158	3,017	2,959
MJSL00000000	SRR1814336	BCW_2355	83	31	3,052,281	3,082	3,025
MJSM00000000	SRR1814337	BCW_2356	97	20	2,964,368	2,981	2,924
MJSN00000000	SRR1814338	BCW_2357	110	23	2,939,864	2,929	2,871
MJSO00000000	SRR1814339	BCW_2359	87	18	2,916,505	2,908	2,848
MJSP00000000	SRR1814340	BCW_2361	151	16	2,945,010	2,933	2,882
MJSQ00000000	SRR1814341	BCW_2362	42	29	3,002,930	3,017	2,955
MJSR00000000	SRR1814342	BCW_2363	136	28	2,993,621	3,023	2,966
MJSS00000000	SRR1814343	BCW_2364	56	39	3,061,043	3,065	3,006
MJST00000000	SRR1814344	BCW_2365	51	24	3,022,563	3,032	2,975
MJSU00000000	SRR1814345	BCW_2367	49	21	2,944,297	2,945	2,881
MJSV00000000	SRR1814346	BCW_2368	44	13	2,930,208	2,922	2,861
MJSW00000000	SRR1814347	BCW_2369	43	21	2,771,135	2,770	2,712
MJSX00000000	SRR1814348	BCW_2370	49	33	3,106,802	3,170	3,112
MJSY00000000	SRR1814349	BCW_2371	38	20	2,957,530	2,963	2,902
MJSZ00000000	SRR1814350	BCW_2372	58	14	2,827,526	2,836	2,783
MJTA00000000	SRR1814351	BCW_2373	56	15	2,995,034	3,016	2,949
MJTB00000000	SRR1814352	BCW_2374	54	18	2,966,392	3,021	2,960
MJTC00000000	SRR1814353	BCW_2375	50	22	2,923,827	2,887	2,826
MJTD00000000	SRR1814354	BCW_2376	42	21	2,813,242	2,846	2,785
MJTE00000000	SRR1814355	BCW_2378	42	35	3,123,182	3,190	3,132
MJTF00000000	SRR1814356	BCW_2379	42	31	3,039,365	3,088	3,030
MJTG00000000	SRR1814357	BCW_2380	37	22	2,918,370	2,900	2,841
MJTH00000000	SRR1814358	BCW_2381	50	22	2,971,254	2,995	2,934
MJTI00000000	SRR1814359	BCW_2382	102	22	3,020,256	3,033	2,974
MJTJ00000000	SRR1814360	BCW_2383	61	25	3,350,928	3,350	3,290
MJTK00000000	SRR1814361	BCW_2384	64	21	2,951,592	2,975	2,914
MJTL00000000	SRR1814362	BCW_2385	55	17	2,996,412	3,017	2,952
MJTM00000000	SRR1814363	BCW_2386	42	23	2,957,654	2,959	2,902
MJTN00000000	SRR1814364	BCW_2387	51	24	3,021,344	3,031	2,973
MJTO00000000	SRR1814365	BCW_2388	54	17	2,889,294	2,870	2,811
MJTP00000000	SRR1814366	BCW_2389	49	11	2,921,087	2,930	2,875
MJTQ00000000	SRR1814367	BCW_2390	165	19	2,886,917	2,864	2,810
MJTR00000000	SRR1814368	BCW_2391	100	20	3,014,266	3,023	2,969
MJTS00000000	SRR1814369	BCW_2392	57	15	2,990,427	3,012	2,954
MJTT00000000	SRR1814370	BCW_2393	92	11	2,853,604	2,834	2,778
MJTU00000000	SRR1814371	BCW_2394	82	21	3,017,853	3,030	2,971
MJTV00000000	SRR1814372	BCW_2395	117	13	2,904,985	2,873	2,821
MJTW00000000	SRR1814374	BCW_2397	95	13	2,908,687	2,916	2,860
MJTX00000000	SRR1814375	BCW_2399	112	21	3,041,356	3,023	2,973
MJTY00000000	SRR1814376	BCW_2400	85	18	2,962,811	2,957	2,898
MJTZ00000000	SRR1814377	BCW_2401	129	13	2,794,214	2,800	2,742
MJUA00000000	SRR1814378	BCW_2402	75	25	3,021,222	3,035	2,976
MJUB00000000	SRR1814379	BCW_2403	74	23	2,962,355	2,972	2,916
MJUC00000000	SRR1814380	BCW_2404	86	13	2,934,172	2,960	2,900
MJCE00000000	SRR1814381	BCW_2405	106	12	2,882,471	2,876	2,818
MJCF00000000	SRR1814382	BCW_2406	94	20	2,898,005	2,866	2,808
MJCG00000000	SRR1814383	BCW_2407	70	22	3,048,640	3,080	3,015
MJCH00000000	SRR1814384	BCW_2408	84	15	2,993,315	3,016	2,961
MJCI00000000	SRR1814385	BCW_2409	67	20	2,977,322	2,999	2,940
MJCJ00000000	SRR1814386	BCW_2411	54	9	2,861,878	2,841	2,781
MJCK00000000	SRR1814387	BCW_2412	59	35	3,129,371	3,196	3,135
MJCL00000000	SRR1814388	BCW_2413	83	27	2,977,946	2,995	2,937
MJCM00000000	SRR1814389	BCW_2414	89	14	2,925,666	2,921	2,860
MJCN00000000	SRR1814390	BCW_2415	75	19	2,941,993	2,921	2,857
MJCO00000000	SRR1814391	BCW_2416	81	18	2,967,434	2,987	2,928
MJCP00000000	SRR1814392	BCW_2417	92	16	2,890,912	2,864	2,807
MJCQ00000000	SRR1814393	BCW_2418	76	21	3,065,940	3,086	3,027
MJCR00000000	SRR1814394	BCW_2419	68	16	2,887,580	2,857	2,791
MJCS00000000	SRR1814395	BCW_2420	52	16	2,943,342	2,942	2,877
MJCT00000000	SRR1814396	BCW_2421	57	21	3,019,802	3,028	2,969
MJCU00000000	SRR1814397	BCW_2422	53	16	2,884,219	2,850	2,788
MJCV00000000	SRR1814398	BCW_2423	96	9	2,934,518	2,923	2,859
MJCW00000000	SRR1814399	BCW_2424	114	16	2,994,257	3,009	2,947
MJCX00000000	SRR1814400	BCW_2425	131	27	2,946,581	2,938	2,872
MJCY00000000	SRR1814455	BCW_2624	92	17	2,936,653	2,909	2,854
MJCZ00000000	SRR1815434	BCW_2984	148	19	2,987,575	3,011	2,953
MJDA00000000	SRR1815435	BCW_2985	134	18	2,989,169	3,010	2,949
MJDB00000000	SRR1815436	BCW_2986	66	14	2,946,854	2,945	2,878
MJDC00000000	SRR1815437	BCW_2987	83	14	2,946,854	2,945	2,878
MJDD00000000	SRR1815438	BCW_2988	89	15	2,941,698	2,940	2,876
MJDE00000000	SRR1815439	BCW_2989	83	24	2,496,910	2,952	2,889
MJDF00000000	SRR1815440	BCW_2990	68	15	2,948,526	2,945	2,879
MJDG00000000	SRR1815441	BCW_2991	93	27	3,007,415	3,021	2,958
MJDH00000000	SRR1815442	BCW_2995	91	31	3,005,169	3,017	2,957
MJND00000000	SRR1815443	BCW_2996	107	25	3,003,951	3,020	2,959
MJNE00000000	SRR1815444	BCW_2997	92	17	2,857,564	2,878	2,807
MJOD00000000	SRR1805510	BCW_3811	68	20	2,977,523	2,972	2,912
MJOE00000000	SRR1805407	BCW_3812	48	12	2,841,618	2,828	2,770
MKMD00000000	SRR1815876	BCW_3813	45	19	2,965,876	2,964	2,903
MKME00000000	SRR1815877	BCW_3814	37	23	3,006,902	3,016	2,955
MKMF00000000	SRR1815878	BCW_3815	35	16	2,842,153	2,843	2,784
MKMG00000000	SRR1815879	BCW_3816	37	20	2,928,884	2,886	2,825
MKMH00000000	SRR1815880	BCW_3817	35	19	2,914,544	2,898	2,839
MKMI00000000	SRR1815881	BCW_3818	28	17	2,878,888	2,862	2,804
MKMJ00000000	SRR1815882	BCW_3819	73	21	2,971,782	2,961	2,900
MKMK00000000	SRR1815883	BCW_3820	40	16	2,868,238	2,857	2,798
MKML00000000	SRR1815884	BCW_3821	31	12	2,859,251	2,837	2,787
MKMM00000000	SRR1815885	BCW_3822	39	28	2,917,738	2,912	2,851
MKMN00000000	SRR1815886	BCW_3823	41	22	2,961,674	2,970	2,907
MKMO00000000	SRR1815887	BCW_3824	52	38	3,009,760	3,034	2,972
MKMP00000000	SRR1815888	BCW_3825	39	14	2,897,778	2,883	2,825
MKMQ00000000	SRR1815889	BCW_3826	64	12	2,892,501	2,860	2,799
MKMR00000000	SRR1815890	BCW_3827	41	17	2,902,289	2,884	2,819
MKMS00000000	SRR1815891	BCW_3828	80	20	2,981,768	3,008	2,950
MKMT00000000	SRR1815892	BCW_3829	33	21	2,867,338	2,828	2,773
MKMU00000000	SRR1815893	BCW_3830	30	12	2,873,745	2,826	2,766
MKMV00000000	SRR1815894	BCW_3831	37	13	2,822,641	2,777	2,716
MKMW00000000	SRR1815895	BCW_3832	32	14	2,885,730	2,863	2,806
MKMX00000000	SRR1815896	BCW_3833	35	25	2,941,985	2,933	2,878
MKMY00000000	SRR1815897	BCW_3834	32	17	2,966,648	2,991	2,935
MKMZ00000000	SRR1815898	BCW_3835	49	10	2,857,933	2,803	2,743
MKNA00000000	SRR1815899	BCW_3836	85	11	2,873,808	2,860	2,800
MKNB00000000	SRR1815900	BCW_3837	49	7	2,903,394	2,872	2,818
MKNC00000000	SRR1815901	BCW_3838	54	10	2,846,174	2,821	2,765
MKND00000000	SRR1815902	BCW_3839	67	19	2,893,770	2,869	2,810
MKNE00000000	SRR1815903	BCW_3840	60	24	2,979,754	2,978	2,921
MKNF00000000	SRR1815904	BCW_3841	68	12	2,852,100	2,838	2,775
MKNG00000000	SRR1815905	BCW_3842	56	11	2,835,578	2,818	2,759
MKNH00000000	SRR1815906	BCW_3843	73	14	2,870,259	2,848	2,792
MKNI00000000	SRR1815907	BCW_3844	83	9	2,918,129	2,921	2,861
MKNJ00000000	SRR1815908	BCW_3845	53	9	2,874,832	2,844	2,784
MKNK00000000	SRR1815909	BCW_3846	315	13	2,927,242	2,918	2,866
MKNL00000000	SRR1815910	BCW_3847	37	12	2,905,480	2,900	2,837
MKNM00000000	SRR1815911	BCW_3848	43	6	2,873,840	2,840	2,780
MKNN00000000	SRR1815912	BCW_3849	52	7	2,838,591	2,802	2,742
MKNO00000000	SRR1815913	BCW_3850	50	15	2,860,446	2,857	2,796
MKNP00000000	SRR1815914	BCW_3851	91	11	2,909,975	2,916	2,860
MKNQ00000000	SRR1815915	BCW_3852	87	8	2,907,378	2,882	2,825
MKNR00000000	SRR1815916	BCW_3853	121	13	2,854,561	2,799	2,746
MKNS00000000	SRR1815917	BCW_3854	237	13	2,858,772	2,797	2,750
MKNT00000000	SRR1815918	BCW_3855	71	7	2,884,000	2,859	2,800
MKNU00000000	SRR1815919	BCW_3856	43	16	2,840,147	2,822	2,762
MKNV00000000	SRR1815920	BCW_3857	82	12	2,918,757	2,941	2,881
MKNW00000000	SRR1815921	BCW_3858	78	15	2,864,782	2,844	2,778
MKNX00000000	SRR1815922	BCW_3859	49	12	2,897,160	2,890	2,833
MKNY00000000	SRR1815923	BCW_3860	49	9	2,844,019	2,805	2,740
MKNZ00000000	SRR1815924	BCW_3861	37	15	2,820,199	2,780	2,719
MKOA00000000	SRR1815925	BCW_3862	25	12	2,870,692	2,822	2,766
MKOB00000000	SRR1815926	BCW_3863	18	39	2,943,002	2,954	2,892
MKOC00000000	SRR1815927	BCW_3864	51	16	2,854,724	2,833	2,773
MKOD00000000	SRR1815928	BCW_3865	29	19	2,926,734	2,927	2,868
MKOE00000000	SRR1815929	BCW_3866	27	21	2,943,390	2,974	2,917
MKOF00000000	SRR1815930	BCW_3867	45	12	2,896,382	2,890	2,829
MKOG00000000	SRR1815931	BCW_3868	41	15	2,891,637	2,891	2,833
MKOH00000000	SRR1815932	BCW_3869	31	16	2,956,706	2,980	2,920
MKOI00000000	SRR1815933	BCW_3870	25	29	2,828,150	2,838	2,788
MKOJ00000000	SRR1815934	BCW_3871	21	37	3,081,204	3,115	3,063
MKOK00000000	SRR1815935	BCW_3872	18	22	2,803,150	2,833	2,775
MKOL00000000	SRR1815936	BCW_3873	27	15	2,837,427	2,820	2,766
MKOM00000000	SRR1815937	BCW_3874	20	60	2,958,576	2,962	2,909
MKON00000000	SRR1815938	BCW_3875	57	18	2,888,109	2,891	2,835
MKOO00000000	SRR1815939	BCW_3876	46	22	2,922,426	2,922	2,863
MKOP00000000	SRR1815940	BCW_3877	31	36	3,087,006	3,125	3,066
MKOQ00000000	SRR1815941	BCW_3878	28	24	2,909,010	2,874	2,819
MKOR00000000	SRR1815942	BCW_3879	18	52	2,874,381	2,842	2,786
MKOS00000000	SRR1815943	BCW_3880	24	49	3,004,145	3,051	2,994
MKOT00000000	SRR1815944	BCW_3881	60	17	2,940,478	2,935	2,874
MKOU00000000	SRR1815945	BCW_3882	47	17	2,910,965	2,882	2,811
MKOV00000000	SRR1815946	BCW_3883	55	22	2,905,998	2,883	2,825
MKOW00000000	SRR1815947	BCW_3884	54	23	2,939,753	2,919	2,853
MKOX00000000	SRR1815948	BCW_3885	39	22	2,950,478	2,953	2,885
MKOY00000000	SRR1815949	BCW_3886	30	28	3,071,194	3,048	2,992
MKOZ00000000	SRR1815950	BCW_3887	32	27	2,940,770	2,909	2,850
MKPA00000000	SRR1815951	BCW_3888	45	19	2,947,241	2,948	2,881
MKPB00000000	SRR1815952	BCW_3889	31	18	2,926,393	2,921	2,860
MKPC00000000	SRR1815953	BCW_3890	59	10	2,856,568	2,827	2,765
MKPD00000000	SRR1815954	BCW_3891	39	20	2,967,252	2,986	2,922
MKPE00000000	SRR1815955	BCW_3892	33	18	2,920,115	2,901	2,844
MKPF00000000	SRR1815956	BCW_3893	30	14	2,849,771	2,835	2,779
MKPG00000000	SRR1815957	BCW_3894	29	12	2,882,875	2,874	2,818
MKPH00000000	SRR1815958	BCW_3895	32	15	2,894,936	2,889	2,833
MKPI00000000	SRR1815959	BCW_3896	60	12	2,970,597	2,955	2,894
MKPJ00000000	SRR1815960	BCW_3897	35	18	2,982,868	3,005	2,943
MKPK00000000	SRR1815961	BCW_3898	51	14	2,894,195	2,861	2,801
MKPL00000000	SRR1815962	BCW_3899	48	13	2,860,355	2,852	2,788
MKPM00000000	SRR1815963	BCW_3900	25	23	2,982,615	2,966	2,911
MKPN00000000	SRR1815964	BCW_3901	48	12	2,841,873	2,831	2,774
MKPO00000000	SRR1815965	BCW_3902	43	14	2,931,100	2,950	2,892
MKPP00000000	SRR1815966	BCW_3903	25	15	2,868,643	2,848	2,797
MKPQ00000000	SRR1815967	BCW_3904	36	37	3,035,356	3,050	2,991
MKPR00000000	SRR1815968	BCW_3905	34	14	2,830,709	2,804	2,749
MKPS00000000	SRR1815969	BCW_3906	37	17	2,878,382	2,865	2,807
MJNF00000000	SRR1815970	BCW_3907	63	17	2,928,223	2,918	2,861
MJNG00000000	SRR1815971	BCW_3908	49	24	3,063,990	3,057	2,999
MJNH00000000	SRR1815972	BCW_3909	40	11	2,839,271	2,817	2,763
MJNI00000000	SRR1815973	BCW_3910	47	17	2,758,147	2,771	2,717
MJNJ00000000	SRR1815974	BCW_3911	63	18	2,926,597	2,906	2,845
MJNK00000000	SRR1815975	BCW_3912	49	15	2,852,829	2,837	2,777
MJNL00000000	SRR1815976	BCW_3913	52	17	2,875,683	2,884	2,826
MJNM00000000	SRR1815977	BCW_3914	48	23	2,921,018	2,928	2,868
MJNN00000000	SRR1815978	BCW_3915	40	20	2,875,515	2,848	2,793
MJNO00000000	SRR1815979	BCW_3916	41	18	2,999,138	2,995	2,941
MJNP00000000	SRR1815980	BCW_3917	72	15	2,837,435	2,824	2,765
MJNQ00000000	SRR1815981	BCW_3918	45	17	2,889,824	2,889	2,832
MJNR00000000	SRR1815982	BCW_3919	36	18	2,914,631	2,912	2,856
MJNS00000000	SRR1815983	BCW_3920	44	21	2,902,374	2,865	2,804
MJNT00000000	SRR1815984	BCW_3921	31	21	2,906,315	2,890	2,835
MJNU00000000	SRR1815985	BCW_3922	56	11	2,878,388	2,880	2,821
MJNV00000000	SRR1815986	BCW_3923	46	20	2,927,260	2,959	2,900
MJNW00000000	SRR1815987	BCW_3924	81	19	2,897,709	2,863	2,799
MJNX00000000	SRR1815988	BCW_3925	86	24	3,014,318	3,024	2,964
MJNY00000000	SRR1815989	BCW_3926	54	24	3,056,181	3,071	3,011
MJNZ00000000	SRR1815990	BCW_3927	60	16	2,890,051	2,869	2,810
MJOA00000000	SRR1815991	BCW_3928	58	23	3,122,409	3,159	3,098
MJOB00000000	SRR1815992	BCW_3929	44	21	2,950,233	2,957	2,898
MJOC00000000	SRR1815993	BCW_3930	44	20	2,941,469	2,924	2,861
MJOF00000000	SRR1815994	BCW_3931	90	15	2,889,246	2,870	2,813
MJOG00000000	SRR1815995	BCW_3932	74	19	2,975,068	2,972	2,911
MJOH00000000	SRR1815996	BCW_3933	68	10	2,822,712	2,787	2,731
MJOI00000000	SRR1815997	BCW_3934	66	10	2,829,488	2,795	2,736
MJOJ00000000	SRR1815998	BCW_3935	28	190	3,149,703	3,194	3,138
MJOK00000000	SRR1805379	BCW_3936	39	17	2,737,049	2,725	2,669
MJOL00000000	SRR1816001	BCW_3938	57	18	2,879,444	2,851	2,790
MJOM00000000	SRR1816002	BCW_3939	64	14	2,878,718	2,887	2,823
MJON00000000	SRR1816003	BCW_3940	41	14	2,859,559	2,835	2,776
MJOO00000000	SRR1816004	BCW_3941	86	26	3,074,403	3,087	3,026
MJOP00000000	SRR1816005	BCW_3942	64	18	2,973,453	2,970	2,911
MJOQ00000000	SRR1816006	BCW_3943	43	19	2,962,794	2,980	2,921
MJOR00000000	SRR1816007	BCW_3944	74	32	3,093,581	3,165	3,104
MJOS00000000	SRR1816008	BCW_3945	57	23	2,959,411	3,001	2,943
MJOT00000000	SRR1816053	BCW_4252	41	18	2,904,951	2,915	2,858
MJOU00000000	SRR1816054	BCW_4253	52	29	3,053,325	3,071	3,012
MJOV00000000	SRR1816055	BCW_4254	77	28	3,104,645	3,137	3,079
MJOW00000000	SRR1816056	BCW_4255	64	23	2,935,300	2,917	2,856
MJOX00000000	SRR1816057	BCW_4256	51	34	3,142,820	3,178	3,119
MJOY00000000	SRR1816058	BCW_4257	66	14	2,935,555	2,923	2,862
MJOZ00000000	SRR1816059	BCW_4258	51	25	3,096,969	3,103	3,044
MJPA00000000	SRR1816060	BCW_4259	68	26	2,980,170	2,966	2,905
MJPB00000000	SRR1816061	BCW_4260	48	34	3,013,184	3,016	2,956
MJPC00000000	SRR1816062	BCW_4261	119	29	3,148,803	3,157	3,101
MJPD00000000	SRR1816063	BCW_4262	39	49	3,159,840	3,175	3,119
MJPE00000000	SRR1816064	BCW_4263	47	35	3,165,508	3,198	3,136
MJPF00000000	SRR1816065	BCW_4264	66	14	2,967,285	2,951	2,890
MJPG00000000	SRR1816066	BCW_4265	41	13	3,002,824	2,998	2,935
MJPH00000000	SRR1816067	BCW_4266	47	19	3,028,588	3,054	2,998
MJPI00000000	SRR1816068	BCW_4267	58	22	3,004,577	3,011	2,952
MJPJ00000000	SRR1816069	BCW_4268	35	34	3,044,813	3,053	3,003
MJPK00000000	SRR1816070	BCW_4269	49	14	2,930,225	2,961	2,904
MJPL00000000	SRR1816071	BCW_4270	37	24	3,057,295	3,107	3,046
MJPM00000000	SRR1816072	BCW_4271	43	17	3,003,458	3,004	2,943
MJPN00000000	SRR1816073	BCW_4272	36	36	3,059,631	3,083	3,024
MJPO00000000	SRR1816074	BCW_4273	47	32	3,056,980	3,069	3,007
MJPP00000000	SRR1816075	BCW_4274	35	29	3,092,169	3,142	3,083
MJPQ00000000	SRR1816076	BCW_4275	53	17	2,970,143	2,969	2,910
MJPR00000000	SRR1816077	BCW_4276	30	20	3,023,906	3,032	2,975
MJPS00000000	SRR1816078	BCW_4277	55	10	2,932,904	2,947	2,887
MJPT00000000	SRR1816079	BCW_4278	66	22	2,969,319	3,003	2,949
MJPU00000000	SRR1816080	BCW_4279	61	16	2,959,605	2,941	2,884
MJPV00000000	SRR1816081	BCW_4280	34	18	2,956,561	2,976	2,920
MJPW00000000	SRR1816082	BCW_4281	41	24	2,999,322	2,990	2,934
MJPX00000000	SRR1816083	BCW_4282	128	19	3,053,557	3,069	3,017
MJPY00000000	SRR1816084	BCW_4283	182	34	2,958,989	2,952	2,899
MJPZ00000000	SRR1816085	BCW_4284	150	36	3,126,895	3,167	3,107
MJQA00000000	SRR1816086	BCW_4285	43	19	2,933,555	2,909	2,848
MJQB00000000	SRR1816088	BCW_4287	94	18	3,004,278	3,013	2,956
MJQC00000000	SRR1816089	BCW_4288	47	29	3,087,349	3,115	3,058
MJQD00000000	SRR1816090	BCW_4289	145	15	2,947,025	2,955	2,900
MJQE00000000	SRR1816091	BCW_4290	70	12	3,001,188	2,998	2,939
MJQF00000000	SRR1816092	BCW_4291	161	20	3,084,559	3,109	3,048
MJQG00000000	SRR1816093	BCW_4292	163	28	3,015,259	3,039	2,984
MJQH00000000	SRR1816094	BCW_4293	53	26	3,011,157	3,015	2,955
MJQI00000000	SRR1816095	BCW_4294	44	39	3,008,725	3,024	2,965
MJQJ00000000	SRR1816096	BCW_4295	30	48	3,080,304	3,114	3,057
MJQK00000000	SRR1816097	BCW_4296	32	14	2,829,770	2,814	2,758
MJQL00000000	SRR1816098	BCW_4297	52	25	3,078,586	3,116	3,056
MJQM00000000	SRR1816099	BCW_4298	162	16	2,985,857	2,976	2,926
MJQN00000000	SRR1816100	BCW_4299	55	20	2,965,573	2,991	2,931
MJQO00000000	SRR1816101	BCW_4300	58	14	2,890,745	2,872	2,814
MJQP00000000	SRR1816102	BCW_4301	72	39	3,129,421	3,198	3,138
MJQQ00000000	SRR1816103	BCW_4302	59	19	2,967,831	2,955	2,896
MJQR00000000	SRR1816105	BCW_4304	52	23	2,981,718	2,976	2,917
MJQS00000000	SRR1816106	BCW_4305	17	31	2,904,001	2,902	2,854
MJQT00000000	SRR1816107	BCW_4306	92	34	3,012,898	3,022	2,963
MJQU00000000	SRR1816108	BCW_4307	70	17	2,979,023	2,982	2,924
MJQV00000000	SRR1816109	BCW_4308	48	30	3,149,424	3,149	3,091
MJQW00000000	SRR1805452	BCW_4309	541	29	2,964,367	2,973	2,925
MJQX00000000	SRR1805386	BCW_4311	82	16	2,990,843	3,032	2,974
MJQY00000000	SRR1805458	BCW_4312	66	17	2,989,606	2,988	2,933
MJQZ00000000	SRR1805418	BCW_4313	130	15	2,844,766	2,828	2,775
MJSH00000000	SRR1805444	BCW_4314	386	21	2,987,238	2,963	2,917
MJRA00000000	SRR2982244	BCW_4315	82	6	2,870,740	2,846	2,785
MJRB00000000	SRR1805544	BCW_4316	85	19	3,061,290	3,084	3,023
MJRC00000000	SRR1816370	BCW_4758	79	16	2,988,627	2,995	2,935
MJRD00000000	SRR1816372	BCW_4760	94	11	2,879,424	2,878	2,824
MJRE00000000	SRR1816373	BCW_4761	89	12	2,951,127	2,969	2,914
MJRF00000000	SRR1816374	BCW_4762	56	18	3,078,289	3,109	3,054
MJRG00000000	SRR1816375	BCW_4763	39	23	3,005,047	3,038	2,977
MJRH00000000	SRR1816376	BCW_4764	84	12	2,953,054	2,969	2,913
MJRI00000000	SRR1816377	BCW_4765	82	12	2,882,576	2,881	2,823
MJRJ00000000	SRR1816378	BCW_4766	82	25	2,906,481	2,882	2,810
MJRK00000000	SRR1816379	BCW_4767	51	16	2,972,582	2,956	2,892
MJRL00000000	SRR1816380	BCW_4768	48	19	2,986,043	3,008	2,952
MJRM00000000	SRR1816381	BCW_4769	54	21	2,937,535	2,904	2,842
MJRN00000000	SRR1816382	BCW_4770	47	21	2,932,710	2,904	2,841
MJRO00000000	SRR1816383	BCW_4771	37	25	2,931,128	2,904	2,843
MJRP00000000	SRR1816384	BCW_4772	52	18	2,978,435	2,976	2,915
MJRQ00000000	SRR1816385	BCW_4773	44	18	2,890,220	2,870	2,810
MJRR00000000	SRR1816386	BCW_4774	35	32	2,939,871	2,963	2,905
MJRS00000000	SRR1816387	BCW_4775	68	17	2,936,512	2,902	2,837
MJRT00000000	SRR1816388	BCW_4776	47	9	2,879,862	2,885	2,825
MJRU00000000	SRR1816389	BCW_4777	48	19	3,005,117	3,025	2,962
MJRV00000000	SRR1816390	BCW_4778	46	14	2,951,95,2	2,973	2,914
MJRW00000000	SRR1816392	BCW_4780	55	15	2,934,901	2,926	2,866
MJRX00000000	SRR1816393	BCW_4781	57	19	3,026,964	3,036	2,974
MJRY00000000	SRR1816394	BCW_4782	42	16	2,941,141	2,949	2,893
MJRZ00000000	SRR1816395	BCW_4783	70	19	2,938,896	2,953	2,895
MJSA00000000	SRR1816396	BCW_4784	64	14	2,954,405	2,961	2,898
MJSB00000000	SRR1816397	BCW_4785	65	20	2,985,984	2,982	2,917
MJSC00000000	SRR1816398	BCW_4786	50	16	2,960,119	2,946	2,884
MJSD00000000	SRR1816399	BCW_4787	42	22	2,980,365	2,984	2,920
MJSE00000000	SRR1816400	BCW_4788	55	19	2,929,400	2,894	2,836
MJSF00000000	SRR1816401	BCW_4789	47	8	2,884,261	2,888	2,831
MJSG00000000	SRR1816402	BCW_4790	44	26	3,008,528	3,038	2,975
Avg			135	20	2,948,523	2,950	2,891

^a^ CDSs, coding sequences.
